# Associations between the morphological parameters of proximal tibiofibular joint (PTFJ) and changes in tibiofemoral joint structures in patients with knee osteoarthritis

**DOI:** 10.1186/s13075-022-02719-8

**Published:** 2022-01-27

**Authors:** Jun Chang, Tianyu Chen, Yizhu Yan, Zhaohua Zhu, Weiyu Han, Yi Zhao, Benny Antony, Anita Wluka, Tania Winzenberg, Flavia Cicuttini, Changhai Ding

**Affiliations:** 1 Clinical Research Centre, Zhujiang Hospital, Southern Medical University, Guangzhou, Guangdong China; 2 Menzies Institute for Medical Research, University of Tasmania, Hobart, Tasmania Australia; 3 Department of Orthopaedics, 4th Affiliated Hospital, Anhui Medical University, Hefei, Anhui China; 4 Department of Orthopedics, The Third Affiliated Hospital of Southern Medical University, Guangzhou, 510000 China; 5 Department of Rheumatology, Xuanwu Hospital, Capital Medical University, Beijing, China; 6 Department of Public Health and Preventive Medicine, Monash University, Melbourne, Australia

## Abstract

**Background:**

To describe the longitudinal associations between the morphological parameters of proximal tibiofibular joint (PTFJ) and joint structural changes in tibiofemoral compartments in patients with knee osteoarthritis (OA).

**Methods:**

The participants were selected from the Vitamin D Effects on Osteoarthritis (VIDEO) study. PTFJ morphological parameters were measured on coronal and sagittal MRI. The contacting area (S) of PTFJ and its projection areas onto the horizontal (load-bearing area, Sτ), sagittal (lateral stress-bolstering area, Sφ), and coronal plane (posterior stress-bolstering area, Sυ) were assessed. Knee structural abnormalities, including cartilage defects, bone marrow lesions (BMLs), and cartilage volume, were evaluated at baseline and after 2 years. Log binominal regression models and linear regression models were used to assess the associations between PTFJ morphological parameters and osteoarthritic structural changes.

**Results:**

In the longitudinal analyses, the S (RR: 1.45) and Sτ (RR: 1.55) of PTFJ were significantly and positively associated with an increase in medial tibial (MT) cartilage defects. The Sτ (β: − 0.07), Sυ (β: − 0.07), and S (β: − 0.06) of PTFJ were significantly and negatively associated with changes in MT cartilage volume. The Sτ (RR: 1.55) of PTFJ was positively associated with an increase in MT BMLs, and Sφ (RR: 0.35) was negatively associated with an increase in medial femoral BMLs.

**Conclusions:**

This longitudinal study suggests that higher load-bearing area of PTFJ could be a risk factor for structural changes in medial tibiofemoral (MTF) compartment in knee OA.

**Trial registration:**

Clinicaltrials.gov Identifier: NCT01176344

Anzctr.org.au Identifier: ACTRN12610000495022

Date of registration: 7 May 2010

**Supplementary Information:**

The online version contains supplementary material available at 10.1186/s13075-022-02719-8.

## Introduction

Knee osteoarthritis (OA) involves tibiofemoral and patellofemoral components [1]. However, little attention has been paid to the proximal tibiofibular joint (PTFJ) and its contribution to knee OA. The PTFJ is a synovial sliding joint characterized by articular cartilage performing a load-bearing function [2].

The medial tibiofemoral compartment of the knee joint is more often affected by OA than the lateral tibiofemoral compartment [3]. This may be because of the biomechanical characteristics of the joint. In physiological conditions, 60–80% of the total intrinsic compressive load transmitted across the knee is on the medial compartment [4]. This loading becomes greater in a knee with varus alignment [5], which may be the cause for or result of progressive knee OA [6]. In a varus knee, the loading-bearing axis is shifted to medial tibiofemoral compartment, so that the medial compartment bears great stress [7]. However, it is still not well understood why mechanical loading is greater in the medial than the lateral tibiofemoral compartment.

Many treatment options have been designed to reduce the load on the medial tibiofemoral compartment, including high tibial osteotomy (HTO) [8], distal femoral varus osteotomy [9], and valgus loading braces [10]. Interestingly, Yang et al. [11] reported that proximal fibular osteotomy could significantly improve the clinical outcomes in patients with medial compartment OA. The fibula is considered one of the most important supporting structures of the leg, which is assumed to bear one-sixth of the leg’s static load in a biostatic mode. Furthermore, this force is mainly transmitted through the PTFJ [2]. The knee joint has three main compartments: patellofemoral, medial tibiofemoral (MTF), and lateral tibiofemoral (LTF). In addition, the synovial joint PTFJ, located between the lateral condyle of the tibia and the head of the fibula, is considered to be the “fourth compartments” of the knee joint. PTFJ has rarely been studied in comparative morphology because it is thought to play a relatively minor role in mechanical loading.

Joint contacting areas are fundamental to the understanding of load transmission through the joint and its relationship to local structural damage in knee OA [12]. Contacting area measurements are useful because they provide information about where loads are transmitted on the cartilage surface [13]. Most studies have focused on the patellofemoral joint [13, 14] and tibiofemoral joint contacting areas [15, 16]. Little or no work has been conducted on the PTFJ contacting areas. Previous studies have measured the morphology of PTFJ from cadaveric knee specimens [17–20]. Eichenblat and Nattan [18] revealed seven PTFJ joint types when assessing PTFJ configuration and reported a statistically significant correlation between the presence of PTFJ OA and knee OA, particularly in the medial tibiofemoral compartment. Recently, we reported that irregular PTFJ joint types were associated with osteoarthritic changes in the lateral compartment in older adults [21]. Based on previous findings, we hypothesized that PTFJ morphological measures were associated with increased risks of osteoarthritic changes in the medial rather than lateral tibiofemoral compartment, and this needs to be validated by further cohort studies.

Therefore, in this study, we aimed to describe the longitudinal associations between the morphological parameters of the PTFJ and joint structural changes in the tibiofemoral compartments in patients with knee OA.

## Methods

### Patients

Participants with knee OA were recruited to the Vitamin D Effects on Osteoarthritis (VIDEO) study, a multi-center, randomized, and double-blind clinical trial to evaluate the effects of vitamin D supplementation in patients with knee OA. Five knees lacked of readable MRI data and were excluded. Baseline data were collected for 408 participants (mean age: 63.2 years, 50% women, 149 from Melbourne and 259 from Tasmania), and 335 (82.1%) completed an approximately 2-years follow-up. In brief, eligible participants had symptomatic knee OA (assessed according to the American College of Rheumatology criteria) [22]. The exclusion criteria included grade 3 radiographic changes according to Altman and Gold’s atlas [23], severe knee pain on standing (> 80 mm on a 100-mm VAS), contraindication to MRI, rheumatoid or psoriatic arthritis, lupus, cancer, and history of significant knee trauma. The Tasmania Health and Human Medical Research Ethics Committee and Monash University Human Research Ethics Committee approved this study. Written informed consent was obtained from all participants. For the purpose of analysis, treatment and placebo groups were combined as a cohort.

### MRI assessment

MRI scans of the knees were performed at baseline and follow-up. Image sequences included the following: (1) a coronal or sagittal T1-weighted, fat-saturated, three-dimensional (3D) spoiled gradient echo with flip angle 30°; repetition time: 40 ms; echo time: 7 ms; acquisition time: 5 min 58 s; slice number: 60; pixel matrix: 512 × 256; and slice thickness, 1.5 mm with no gap; and (2) a T2-weighted, fat-saturated, fast spin echo with flip angle 90°; repetition time: 3060 ms; echo time: 94 ms; slice number: 46; pixel matrix: 256 × 224; and slice thickness: 2 mm with no gap.

#### The morphological parameters of PTFJ

**Fig. 1:**
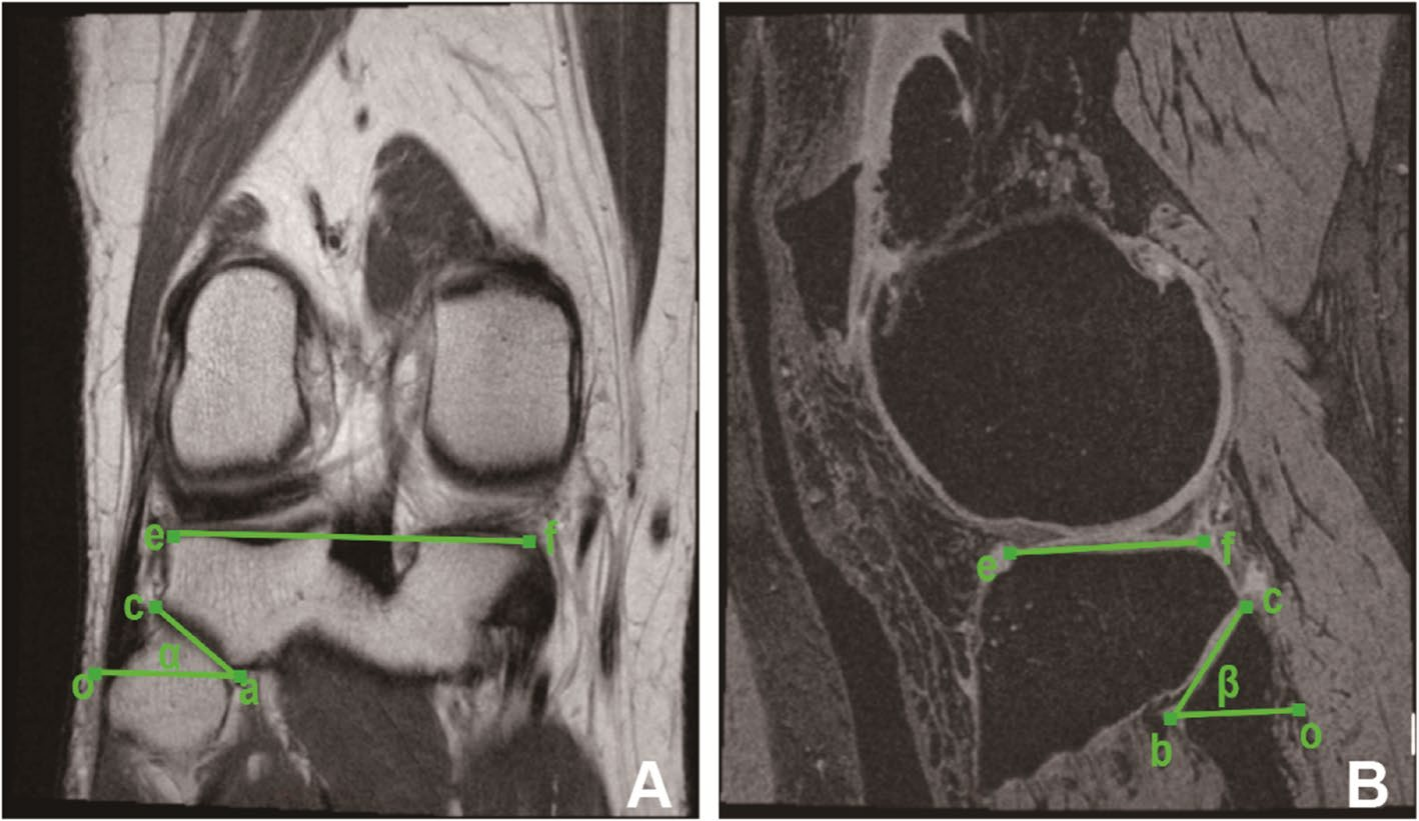
Measures of proximal tibiofibular joint (PTFJ) morphological parameters. **A** T1-weighted coronal MRI slices of the knee. Point *c* is the outermost point of the fibular articular surface, and *a* is the innermost point of the articular surface in PTFJ. *e* is the lateral vertex of the tibial plateau, and *f* is the medial vertex of the tibial plateau. Line *oa* is drawn through *a*, which is parallel to line *ef*. The length of *ac* and the angle *oac* (α) are measured in each slice of MRI. **B** Sagittal fat-suppressed T1-weighted 3D gradient-echo MRI. Point *b* is the lowest point of the fibular articular surface, and *c* is the highest point of the fibular articular surface in PTFJ. *e* and *f* are points that connect the anterior and posterior horns of the lateral meniscus with the tibial plateau, respectively, and line *ef* is parallel to the tibial plateau. Line *ob* is drawn through *b* and is parallel to *ef*. The length between *bc* and the angle of *obc* (β) are then measured in each slice of MRI

The morphological status of PTFJ was assessed as follows (Fig. 1), by using the software program Osiris. In coronal MRI, point *c* is the outermost point of the fibular articular surface, and *a* is the innermost point of the articular surface in PTFJ. *e* is the lateral vertex of the tibial plateau, and *f* is the medial vertex of the tibial plateau. Line *oa* is drawn through *a*, which is parallel to line *ef*. The length of *ac* and the angle *oac* (*α*) were measured at each slice of MRI. In sagittal MRI from the same participant, point *b* is the lowest point of the fibular articular surface, and *c* is the highest point of the fibular articular surface in PTFJ. *e* and *f* are points that connect the anterior and posterior horns of the lateral meniscus with the tibial plateau, respectively, and line *ef* runs parallel to the tibial plateau. Line *ob* is drawn through *b* and is parallel to *ef*. The length between *bc* and the angle of *obc* (*β*) was then measured at each slice of MRI. Next, we calculated the average angles of PTFJ in both coronal (Ave_COR_ang) and sagittal (Ave_SAG_ang) planes, contacting area of PTFJ based to the ideal model (marked as “S”), load-bearing area (“Sτ”), lateral stress-bolstering area (“Sφ”), and posterior stress-bolstering area (“Sυ”) of PTFJ (cm^2^). This method was valid and reliable for assessing the PTFJ and could be used to measure PTFJ morphology in knee OA. Intra-observer and inter-observer correlation coefficients were validated for the measurements of morphological parameters of PTFJ previously. PTFJ typing measures were repeated by one reader (JC) 3 months later or scored by two readers (JC and TM) independently in 50 randomly selected participants to calculate intra-reader and inter-reader reliabilities. The intraclass correlation coefficients (ICCs) were 0.90–0.95, and the inter-reader reliabilities were 0.90–0.94 [24].

#### Cartilage defects

The cartilage defects (0–4) were assessed at the medial tibial, medial femoral, lateral tibial, and lateral femoral sites using T1-weighted images as previously described and were further confirmed using T2-weighted images as follows [24]: grade 0, normal cartilage; grade 1, focal blistering and intra-cartilaginous low-signal intensity area with an intact surface and bottom; grade 2, irregularities on the surface or bottom and loss of thickness < 50%; grade 3, deep ulceration with loss of thickness > 50%; and grade 4, full-thickness chondral wear with exposure of subchondral bone. The intra- (ICCs: 0.89–0.94) and inter-observer reliabilities (ICC: 0.85–0.93) were excellent [25]. Increase in cartilage defects was calculated as follows: cartilage defects change = follow-up cartilage defects–baseline cartilage defects.

#### Bone marrow lesions (BMLs)

BMLs were defined as discrete areas of increased signal adjacent to the subcortical bone. The areas were measured semi-quantitatively using a modified Whole-Organ Magnetic Resonance Imaging Score (WORMS) method in 15 sub-regions [26]. The medial compartment was divided into three subregions (anterior, central, and posterior), and BMLs were scored categorically according to the maximal percentage of bone area that the lesion occupied within the total subregion. We scored grade 0 if there were no BMLs, grade 1 for lesions occupying < 25% of the subregion, grade 2 for lesions occupying between 25 and 50% of the subregion, and grade 3 for lesions occupying > 50% of the subregion. The final score was calculated as the total of subregional scores. The BMLs scores (0–9) were assessed at the medial tibial, medial femoral, lateral tibial, and lateral femoral sites. The intra- and inter-observer reliability of this BMLs scoring system has been shown to be excellent [27]. Increase in BMLs was calculated as follows: bone marrow lesions change = follow-up BMLs–baseline BMLs.

#### Cartilage volume

Knee cartilage volume was determined on T1-weighted MR images with image processing on an independent workstation, as previously described [25]. The tibial cartilage volumes were calculated by manually drawing disarticulation contours around the cartilage boundaries, section by section, which were then resampled for the final 3D rendering using OsiriX imaging software. The intra-class correlation coefficients ranged from 0.92 to 0.96 for intra-observer reliabilities. Changes in cartilage volume per annum in each site were calculated as shown: (follow-up cartilage volume–baseline cartilage volume)/(baseline cartilage volume × follow-up time); absolute change (mL) = follow-up volume–baseline volume.

#### Knee tibial plateau bone area

The areas of medial and lateral tibial plateau bone were measured manually on the three slices on axial T1-weighted MR images closest to the tibial cartilage. An average of these three areas was used as an estimate of the tibial plateau bone area.

### Anthropometrics measures

Height (cm) was measured to the nearest 0.1 cm (with shoes removed) using a stadiometer (Leicester Height Measure, Invicta Plastics Ltd, Leicester, UK). Weight (kg) was measured to the nearest 0.1 kg using an electronic scale, with bulky clothing removed. Body mass index (BMI) [28] was calculated from these data as follows: weight (kg)/height^2^ (m^2^).

### Statistical analyses

Baseline characteristics were compared between participants with high and low load-bearing area of PTFJ using Student’s *t*-tests for continuous data or Chi square tests for proportions.

Log binominal regression analyses were used to assess associations between PTFJ morphological parameters (independent variable) and increases in cartilage defects or BMLs (dependent variables) before and after adjustment for age, sex, height, weight, tibial plateau bone area, radiographic osteoarthritis (ROA), and intervention. Relative risks (RRs) were estimated using these analyses where the outcome was dichotomous.

The longitudinal associations between the morphological parameters of PTFJ and the change of tibial cartilage volume were analyzed using linear regression analyses with adjustment for age, sex, height, weight, ROA, tibial plateau bone area, and intervention. Scatter plots were used to examine the associations between baseline load-bearing area of PTFJ and changes in cartilage volume (including medial and lateral tibial) per annum.

All statistical analyses were performed on Stata V.13.0 (Stata Corp., College Station, TX, USA), and *P <* 0.05 was considered as statistical significance.

## Results

### Participants

A total of 408 subjects aged between 49 and 80 years (mean: 63.2 years) participated in our study (Table 1). Of these, 357 participants completed the longitudinal study. There were no significant differences in demographic factors (age, sex, and BMI) between these participants and those who were lost to follow-up (*n* = 51; data are shown in Supplementary Table 1). The mean load-bearing area (“Sτ”) of PTFJ was 1.81 cm^2^ (SD: 0.61, range: 0.53–4.08). Participants were grouped into two groups (high or low load-bearing area) based on the median of the load-bearing area (1.68 cm^2^), in order to make the characteristics more clearly displayed. Those with high load-bearing area had greater height, weight, medial tibiofemoral (MTF) joint space narrowing (JSN), MTF cartilage defect score, MTF BMLs score, and medial tibial cartilage volume than those with low load-bearing area of PTFJ. The proportion of male patients was higher among those with high load-bearing area of PTFJ. There were no significant differences in MTF osteophytes, LFT osteophytes, LTF cartilage defects, LTF BMLs, and lateral tibial cartilage volume at baseline between the two groups.

**Table 1:** Characteristics of participants

Characteristics	Low load-bearing area *n* = 204	High load-bearing area *n* = 204	*P* value
Age, years	62.9 ± 7.0	63.5 ± 7.2	0.37
Female (%)	138 (67.6%)	67 (32.8%)	<0.001
Height, cm	165.3 ± 9.0	171.7 ± 9.2	<0.001
Weight, kg	81.8 ± 16.4	86.5 ± 14.9	0.003
Tibial bone area, cm^2^	30.6 ± 4.6	34.8 ± 5.2	<0.001
ROA (0–30)	7.2 ± 5.0	7.4 ± 5.1	0.74
MTF cartilage defects (0–8)	4.3 ± 1.8	5.2 ± 2.3	<0.001
LTF cartilage defects (0–8)	4.3 ± 1.7	4.2 ± 1.9	0.33
MTF BMLs (0–18)	0.8 ± 1.6	1.9 ± 2.6	<0.001
LTF BMLs (0–18)	0.9 ± 1.4	0.9 ± 1.3	0.97
MT cartilage volume	1.4 ± 0.4	1.6 ± 0.4	<0.001
LT cartilage volume	2.0 ± 0.7	2.1 ± 0.7	0.08

Participants were grouped into two groups (high or low load-bearing area) based on the median of the load-bearing area (1.68 cm^2^). Results are shown as frequency (%) or mean (SD)

*Abbreviations*: *ROA* Radiographic OA, *MTF* Medial tibiofemoral, *LTF* Lateral tibiofemoral, *BMLs* Bone marrow lesion, *MT* Medial tibial, *LT* Lateral tibial

### PTFJ morphology and increases in tibiofemoral cartilage defects

Longitudinal associations between the morphological parameters of PTFJ and increases in the MTF cartilage defects are presented in Table 2. In the multivariable analyses, S and Sτ of PTFJ were significantly and positively associated with an increase in medial tibial cartilage defects over 2 years, after adjustment for age, sex, height, weight, ROA, tibial plateau bone area, and intervention. Ave_COR_ang, Ave_SAG_ang, Sφ, and Sυ were not significantly associated with an increase in medial tibial cartilage defects over 2 years. There were no significant associations between PTFJ morphological parameters and an increase in medial femoral cartilage defects. In addition, PTFJ morphological parameters were not significantly associated with increases in cartilage defects in the lateral tibiofemoral compartments (Supplementary Table 2).

**Table 2:** Longitudinal associations between the morphological parameters of PTFJ and increases in medial tibiofemoral cartilage defects

	Univariable	Multivariable^a^
	RR (95% CI)	RR (95% CI)
*Increase in medial tibial cartilage defects*		
**Ave_COR_ang**	0.997 (0.992, 1.002)	0.998 (0.991, 1.005)
**Ave_SAG_ang**	1.002 (0.994,1.010)	1.001 (0.993, 1.009)
**S**	1.242 (0.950, 1.626)	**1.445 (1.025, 2.037)**
**Sτ**	1.329 (0.926, 1.836)	**1.554 (1.053, 2.294)**
**Sφ**	0.953 (0.804, 1.130)	0.909 (0.767, 1.107)
**Sυ**	1.056 (0.965, 1.153)	1.008 (0.886, 1.147)
*Increase in medial femoral cartilage defects*		
**Ave_COR_ang**	0.998 (0.993,1.004)	0.999 (0.993,1.005)
**Ave_SAG_ang**	0.992 (0.983,1.001)	0.994 (0.986,1.003)
**S**	1.005 (0.934, 1.082)	0.993 (0.902,1.092)
**Sτ**	**1.108 (1.027,1.216)**	1.020 (0.918,1.132)
**Sφ**	1.005 (0.855,1.181)	0.991 (0.821, 1.196)
**Sυ**	0.970 (0.865, 1.087)	1.016 (0.918, 1.194)

*Abbreviations*: PTFJ, proximal tibiofibular joint; Ave_COR_ang, the average angles of PTFJ in coronal plane; Ave_SAG_ang, the average angles of PTFJ in sagittal plane; S, contacting area of PTFJ; Sτ, load-bearing area of PTFJ; Sφ, lateral stress-bolstering area of PTFJ; Sυ, posterior stress-bolstering area of PTFJ; ROA, radiographic osteoarthritis

^a^Adjusted for age, sex, height, weight, tibial plateau bone area, ROA, and intervention

### PTFJ morphology and change in tibial cartilage volume

**Fig. 2:**
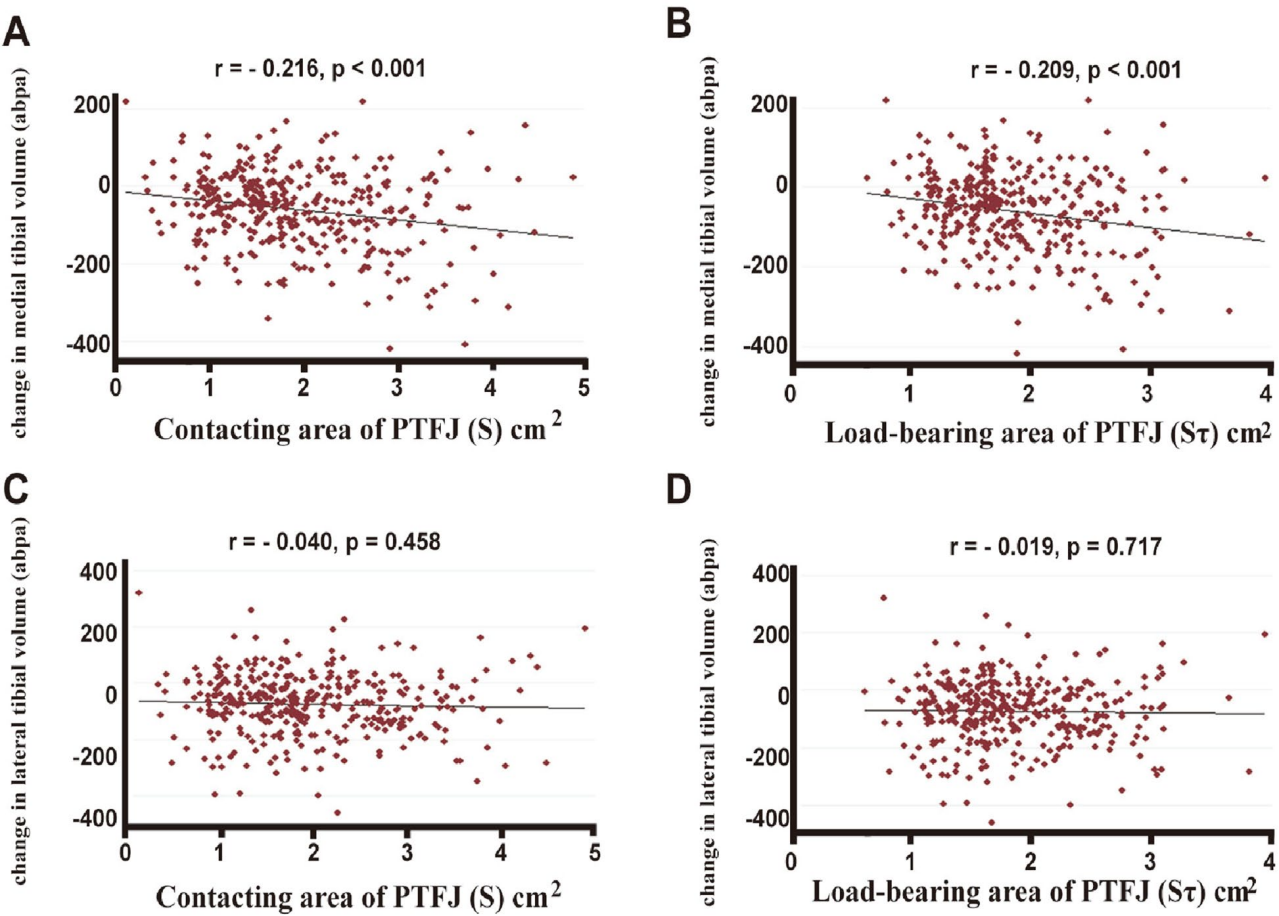
Associations between the areas of PTFJ and change in tibial cartilage volume per annum. The contacting area and load-bearing area of PTFJ were significantly associated with increased loss of cartilage volume at the medial (**A**, **B**) but not the lateral (**C**, **D**) tibial site. Partial *r* and *p* values were obtained after adjustment for age, sex, height, weight, and total tibial bone size

Figure 2 and Table 3 show the longitudinal relationships of PTFJ morphology with tibial cartilage volume. The Sτ of PTFJ was negatively associated with the absolute change in medial tibial cartilage volume over time (Fig. 2A, B). In linear regression analyses, the Sτ, Sυ, and S of PTFJ were significantly and negatively associated with change in medial tibial cartilage volume. In contrast, PTFJ morphological parameters were not significantly associated with change in lateral tibial cartilage volume (Supplementary Table 3, Fig. 2C, D).

**Table 3:** Longitudinal associations between the morphological parameters of PTFJ and changes in medial tibial cartilage volume

	Univariable	Multivariable^a^
	***β*** (95% CI)	***β*** (95% CI)
*Change of medial tibial cartilage volume*		
**Ave_COR_ang**	0.001 (− 0.001, 0.004)	0.001 (− 0.001, 0.003)
**Ave_SAG_ang**	0.001 (− 0.002, 0.004)	0.002(− 0.001, 0.005)
**S**	**− 0.061(− 0.089, − 0.033)**	**− 0.062(− 0.097, − 0.027)**
**Sτ**	**− 0.070 (− 0.104, − 0.037)**	**− 0.070(− 0.108, − 0.029)**
**Sφ**	0.030(− 0.037, 0.103)	0.050(− 0.021,0.121)
**Sυ**	**− 0.076(− 0.119, − 0.034)**	**− 0.066(− 0.119, − 0.014)**

*Abbreviations*: PTFJ, proximal tibiofibular joint; Ave_COR_ang, the average angles of PTFJ in coronal plane; Ave_SAG_ang, the average angles of PTFJ in sagittal plane; S, contacting area of PTFJ; Sτ, load-bearing area of PTFJ; Sφ, lateral stress-bolstering area of PTFJ; Sυ, posterior stress-bolstering area of PTFJ; ROA, radiographic osteoarthritis

^a^Adjusted for age, sex, height, weight, tibial plateau bone area, ROA, and intervention

### PTFJ morphology and increases in tibiofemoral BMLs

Table 4 shows the longitudinal associations between PTFJ morphological parameters and increases in the MTF BMLs. In multivariable analyses, the Sτ of PTFJ was positively associated with an increase in medial tibial BMLs, and Sφ was negatively associated with an increase in medial femoral BMLs. No other morphological parameter of PTFJ was associated with an increase in lateral, medial, tibial, or femoral BMLs (Supplementary Table 4).

**Table 4:** Longitudinal associations between the morphological parameters of PTFJ and increases in medial tibiofemoral BMLs

	Univariable	Multivariable^a^
	RR (95% CI)	RR (95% CI)
*Increase in medial tibial BMLs*		
**Ave_COR_ang**	0.994 (0.914, 1.028)	0.997 (0.923,1.030)
**Ave_SAG_ang**	0.989 (0.949, 1.032)	0.985 (0.945, 1.028)
**S**	**1.539 (1.172, 2.021)**	1.400 (0.979, 2.003)
**Sτ**	**1.705 (1.232, 2.358)**	**1.547 (1.029, 2.328)**
**Sφ**	0.808 (0.429, 1.709)	0.803 (0.480, 1.392)
**Sυ**	1.184 (0.728, 1.927)	0.945 (0.519,1.722)
*Increase in medial femoral BMLs*		
**Ave_COR_ang**	0.975 (0.949, 1.002)	0.974 (0.947, 1.002)
**Ave_SAG_ang**	0.987 (0.949, 1.026)	0.985 (0.947, 1.026)
**S**	1.015 (0.738, 1.396)	1.040 (0.697, 1.552)
**Sτ**	1.091 (0.751, 1.586)	1.152 (0.736, 1.804)
**Sφ**	**0.381 (0.159, 0.913)**	**0.352 (0.144, 0.864)**
**Sυ**	1.073 (0.599, 1.508)	1.069 (0.529, 1.766)

*Abbreviations*: PTFJ, proximal tibiofibular joint; BMLs, bone marrow lesions; Ave_COR_ang, the average angles of PTFJ in coronal plane; Ave_SAG_ang, the average angles of PTFJ in sagittal plane; S, contacting area of PTFJ; Sτ, load-bearing area of PTFJ; Sφ, lateral stress-bolstering area of PTFJ; Sυ, posterior stress-bolstering area of PTFJ; ROA, radiographic osteoarthritis

^a^Adjusted for age, sex, height, weight, tibial plateau bone area, ROA, and intervention

## Discussion

To the best of our knowledge, this is the first longitudinal study to demonstrate the associations between PTFJ morphology and changes in tibiofemoral compartment structures in patients with knee OA. Our major findings were that higher load-bearing area of PTFJ at baseline was associated with increasing medial tibial cartilage defects, BMLs, and cartilage volume loss over 24 months after adjustment for potential confounding factors. These associations were statistically significant in the medial, but not lateral tibiofemoral compartment. These results suggest that PTFJ morphology may have effects on disease progression in medial tibiofemoral compartment in patients with knee OA, and higher load-bearing area of PTFJ could be a risk factor for the progression of medial tibiofemoral OA.

There is a paucity of literature concerning the clinical significance of the PTFJ in knee OA. PTFJ is composed of the tibial facet joint located at the posterolateral tibial condyle and the fibular facet joint on the medial and upper surface of the fibular head. The fibula is considered to be a supporting structure during weight-bearing process. Therefore, the PTFJ was believed to have compressive function [2], but Ogden [17] suggests that the PTFJ mainly bears a tensional force rather than a compressive one. In addition to rotational movement of the fibula relative to the tibia, the fibular head moves in the anterior-posterior plane as a function of knee flexion. Scott et al. [29] demonstrated that the anatomical variations of PTFJ had effects on PTFJ translation, further affecting tibiofemoral loading in human specimens. Burkhart et al. [20] measured PTFJ morphology including inclination angle, surface areas, articular surface concavity and shape from embalmed specimens. Regression analyses were performed to determine the relationships between PTFJ morphology and PTFJ kinematics, which showed that PTFJ morphology likely had an effect on tibiofemoral loading. In a knee with varus malalignment, this load-bearing line passes medial to the center of the knee, and an abduction moment arm is created, which increases compressive force across the medial compartment. In the presence of existing knee OA, varus malalignment has been shown to lead to a 4-fold increase in medial tibiofemoral knee OA progression [6]. Therefore, PTFJ morphology might influence joint structures in MTF compartment in patients with knee OA.

In the current study, we measured the inclination angle of PTFJ from coronal and sagittal MRI. The inclination angle was not statistically correlated with changes in the tibiofemoral structures in the longitudinal analysis. We further measured the PTFJ articular surface (S) [24]. The S of PTFJ was positively correlated with the increasing medial tibial cartilage defects and negatively correlated with the medial tibial cartilage volume. To analyze the biomechanics of PTFJ, we conducted stress analysis of PTFJ in the static load and calculated posterior stress-bolstering area (Sυ), lateral stress-bolstering area (Sφ), and load-bearing area (Sτ) of PTFJ. With every 1 cm^2^ increase in Sτ of PTFJ, tibial cartilage volume decreased by 2.0% per annum (or loss 2.0% per annum) in the medial tibial site. Sφ was the projection area of S onto the sagittal plane that was perpendicular to the coronal plane and was proportional to the lateral force. Sφ bears lateral stress-bolstering force of the fibula from the tibia in inward direction; with inward force increasing, the alignment axis moves from the center to the lateral side, decreasing the force across the medial tibiofemoral compartment. With the upward and forward force increasing, the greater load is shifted to the medial tibia plateau, increasing the force across medial tibiofemoral compartment. Based on these facts, we proposed that the role of Sφ might be opposite to that of Sτ and Sυ in the knee joint [24]. Indeed, we found Sφ was negatively associated with an increase in medial femoral BMLs in this sample suggesting a beneficial effect. BMLs changes could be detected in the short term in knee OA [30, 31], and this could explain why we only found a significant association of Sφ with BMLs. Further studies are required to confirm these findings.

Uneven lateral support of tibial plateau from fibula is a key factor that leads to the non-uniform settlement of the bilateral plateau and the shift of the mechanical axis to the MTF compartment, resulting in osteoarthritic degradation in MTF compartment [11]. We did not find significant associations between baseline PTFJ morphology and changes in lateral tibial cartilage volume or defects and BMLs. This suggests that PTFJ has no significant effects on structural changes in lateral tibiofemoral compartment in patients with knee OA.

The relationship between load-bearing area of PTFJ and the natural progression of primary knee OA has not previously been demonstrated. Theories regarding this relationship have been based on biomechanical models and surgical outcome studies. Two recent orthopedic reports showed that proximal fibular osteotomy could relieve knee pain and improve medial joint space and function in patients with medial knee OA [11, 32]. Commonly used methods of proximal fibular osteotomy in clinic were resect a 2-cm segment of the fibula 6–10 cm below the fibular head. A recent study reported that after proximal fibular osteotomy, joint space improvement was observed in MTF compartment, and there was a statistically significant reduction of pressure on the medial condyle. The aim of this procedure is to shift the mechanical axis of the lower limb from the medial to the lateral compartment, thereby reducing the load and contact area over the medial compartment. This suggests that targeting PTFJ would have beneficial effects on the medial tibiofemoral OA.

Our study has several potential limitations. First, we measured the load-bearing area of PTFJ, which is a virtually invisible surface. However, to analyze the biomechanics of PTFJ, the load-bearing area of PTFJ is the projection of the PTFJ contacting surface onto the horizontal plane, which is proportional to the load bearing, and the method we used is validated and its reproducibility was high, suggesting this was not a major issue. Second, the VIDEO study was originally designed as an RCT to examine the effect of vitamin D supplementation rather than as a cohort study in patients with knee OA; therefore, the results could be affected by the intervention. However, all associations from longitudinal analyses remained significant after adjustment for the intervention. Third, as a number of analyses have been performed, multiple testing might be a concern. However, multiplicity corrections are not necessary for the exploratory study as this does not need pre-specified hypotheses and includes typically large numbers of data-generated hypothesis tests [33]. Moreover, leg malalignment would affect the association of PTFJ with structural changes in medial MTF compartment; however, in the original VIDEO study, we did not measure leg malalignment. This needs to be explored in the future cohort studies. Finally, these findings are from studies of participants with preexisting knee OA. Although the load-bearing area of PTFJ may be a potent risk factor for medial knee OA progression and/or a useful clinical marker of increasing disease severity, it has not yet been shown to be a risk factor for incident medial knee OA.

## Conclusions

This longitudinal study suggests that higher load-bearing area of PTFJ could be a risk factor for the progression of medial tibiofemoral OA.

## Supplementary Information

**Additional file 1: Table S1.** Comparison of baseline characteristics of participants who did and did not complete the study.

**Additional file 2: Table S2.** Longitudinal associations between the morphological parameters of PTFJ and increases in lateral tibiofemoral cartilage defects.

**Additional file 3: Table S3.** Longitudinal associations between the morphological parameters of PTFJ and changes in lateral tibial cartilage volume.

**Additional file 4: Table S4.** Longitudinal associations between the morphological parameters of PTFJ and increases in tibiofemoral BMLs.

## Data Availability

The data used to support the findings of this study are available from the corresponding author upon request.

## Abbreviations

PTFJ: Proximal tibiofibular joint
MTF: Medial tibiofemoral
LTF: Lateral tibiofemoral
BMLs: Bone marrow lesions
MT: Medial tibial
LT: Lateral tibial
OA: Osteoarthritis
S: Contacting area of PTFJ
Sτ: Load-bearing area of PTFJ
Sφ: Lateral stress-bolstering area of PTFJ
Sυ: Posterior stress-bolstering area of PTFJ
ROA: Radiographic osteoarthritis
Ave_COR_ang: The average angles of PTFJ in coronal plane
Ave_SAG_ang: The average angles of PTFJ in sagittal plane
VIDEO: Vitamin D Effects on Osteoarthritis
HTO: High tibial osteotomy
WORMS: Whole-Organ Magnetic Resonance Imaging Score
BMI: Body mass index
RRs: Relative risks
JSN: Joint space narrowing
