# Supplementary material for: Associations between the morphological parameters of proximal tibiofibular joint (PTFJ) and changes in tibiofemoral joint structures in patients with knee osteoarthritis
Source: Arthritis Res Ther. 2022 Jan 27;24:34. doi: 10.1186/s13075-022-02719-8 (PMC8793191; doi:10.1186/s13075-022-02719-8)
Supplement: Supplementary file 1 — Additional file 1: Table S1. Comparison of baseline characteristics of participants who did and did not complete the study. [file 13075_2022_2719_MOESM1_ESM.docx]

**Supplementary Table 1.** Comparison of baseline characteristics of participants who did and did not complete the study

| Characteristics | Completed (n=357) | Not completed (n=51) | *p* Value |
| --- | --- | --- | --- |
|  | n(%) | |  |
| Age, years | 63.2± 7.1 | 63.2±6.9 | 0.98 |
| Female (%) | 178(49.9%) | 27(52.9%) | 0.68 |
| Height, cm | 168.8±9.6 | 165.9±9.4 | 0.05 |
| Weight, kg | 84.3±15.6 | 82.8±17.5 | 0.50 |
| Tibial bone area, cm^2^ | 32.9±5.4 | 31.8±5.2 | 0.20 |
| ROA (0–30) | 7.4±5.1 | 6.9±5.8 | 0.57 |
| MTF cartilage defects (0–8) | 4.8±2.1 | 4.5±2.3 | 0.32 |
| LTF cartilage defects (0–8) | 4.3±1.8 | 4.0±1.8 | 0.28 |
| MTF BMLs (0–18) | 1.4±2.3 | 1.3±2.0 | 0.93 |
| LTF BMLs (0–18) | 0.9±1.4 | 1.0±0.6 | 0.47 |
| MT cartilage volume | 1.5±0.5 | 1.4±0.5 | 0.07 |
| LT cartilage volume | 2.1± 0.7 | 2.0±0.8 | 0.40 |

The results were shown as frequency (%) or mean (SD).

**Abbreviations:**

ROA, radiographic OA; MTF, medial tibiofemoral; LTF, lateral tibiofemoral; BMLs, bone marrow lesion; MT, medial tibial; LT, lateral tibial.
