# Supplementary material for: Associations between the morphological parameters of proximal tibiofibular joint (PTFJ) and changes in tibiofemoral joint structures in patients with knee osteoarthritis
Source: Arthritis Res Ther. 2022 Jan 27;24:34. doi: 10.1186/s13075-022-02719-8 (PMC8793191; doi:10.1186/s13075-022-02719-8)
Supplement: Supplementary file 2 — Additional file 2: Table S2. Longitudinal associations between the morphological parameters of PTFJ and increases in lateral tibiofemoral cartilage defects. [file 13075_2022_2719_MOESM2_ESM.docx]

**Supplementary Table 2.** Longitudinal associations between the morphological parameters of PTFJ and increases in lateral tibiofemoral cartilage defects

|  | **Univariable** | **Multivariable*** |
| --- | --- | --- |
|  | **RR (95% CI)** | **RR (95% CI)** |
| *Increase in lateral tibial cartilage defects* | |  |
| **Ave_COR_ang** | 1.001 (0.975, 1.027) | 1.003 (0.977, 1.029) |
| **Ave_SAG_ang** | 0.987 (0.950, 1.026) | 0.987 (0.948, 1.027) |
| **S** | 0.916 (0.659, 1.281) | 0.786 (0.553, 1.182) |
| **Sτ** | 0.963(0.649,1.429) | 0.847 (0.554, 1.343) |
| **Sφ** | 1.105 (0.849, 1.859) | 1.131 (0.741, 1.539) |
| **Sυ** | 0.828 (0.498, 1.377) | 0.659 (0.359, 1.211) |
| *Increase in lateral femoral cartilage defects* | | |
| **Ave_COR_ang** | 1.011(0.981,1.042) | 1.011(0.981,1.043) |
| **Ave_SAG_ang** | **1.109(1.083, 1.178)** | 1.030(0.983,1.080) |
| **S** | 0.911(0.612, 1.356) | 0.877 (0.538,1.430) |
| **Sτ** | 0.822(0.507,1,334) | 0.771(0.438,1.356) |
| **Sφ** | 1.348(0.536,3.390) | 1.410(0.544, 3.653) |
| **Sυ** | 1.061(0.593, 1.897) | 0.974(0.539, 1.527) |

***Adjusted for age, sex, height, weight, tibial plateau bone area, ROA, and intervention.**

**Abbreviations:**

PTFJ, proximal tibiofibular joint; Ave_COR_ang, the average angles of PTFJ in coronal plane; Ave_SAG_ang, the average angles of PTFJ in sagittal plane; S, contacting area of PTFJ; Sτ, load-bearing area of PTFJ; Sφ, lateral stress-bolstering area of PTFJ; Sυ, posterior stress-bolstering area of PTFJ; ROA, radiographic osteoarthritis.
