# Supplementary material for: Associations between the morphological parameters of proximal tibiofibular joint (PTFJ) and changes in tibiofemoral joint structures in patients with knee osteoarthritis
Source: Arthritis Res Ther. 2022 Jan 27;24:34. doi: 10.1186/s13075-022-02719-8 (PMC8793191; doi:10.1186/s13075-022-02719-8)
Supplement: Supplementary file 3 — Additional file 3: Table S3. Longitudinal associations between the morphological parameters of PTFJ and changes in lateral tibial cartilage volume. [file 13075_2022_2719_MOESM3_ESM.docx]

**Supplementary Table 3.** Longitudinal associations between the morphological parameters of PTFJ and changes in lateral tibial cartilage volume

|  | **Univariable** | **Multivariable*** | | | |
| --- | --- | --- | --- | --- | --- |
|  | **β (95% CI)** | **β (95% CI)** | | | |
| *Change of lateral tibial cartilage volume* |  | |  |  |  |
| **Ave_COR_ang** | -0.001 (-0.003, 0.002) | | -0.001 (-0.004, 0.001) | |  |
| **Ave_SAG_ang** | -0.003(-0.006, 0.001) | | -0.003(-0.007, 0.001) | |  |
| **S** | **-**0.015(-0.046, 0.017**)** | | 0.012(-0.027, 0.051) | |  |
| **Sτ** | **-**0.009(-0.046, 0.028) | | 0.022(-0.021, 0.066**)** | |  |
| **Sφ** | -0.011(-0.089, 0.066) | | -0.002 (-0.067, 0.111) | |  |
| **Sυ** | -0.027(-0.074, 0.020) | | 0.006 (-0.052, 0.065) | |  |

***Adjusted for age, sex, height, weight, tibial plateau bone area, ROA, and intervention.**

**Abbreviations:**

PTFJ, proximal tibiofibular joint; Ave_COR_ang, the average angles of PTFJ in coronal plane; Ave_SAG_ang, the average angles of PTFJ in sagittal plane; S, contacting area of PTFJ; Sτ, load-bearing area of PTFJ; Sφ, lateral stress-bolstering area of PTFJ; Sυ, posterior stress-bolstering area of PTFJ; ROA, radiographic osteoarthritis.
