# Supplementary material for: Associations between the morphological parameters of proximal tibiofibular joint (PTFJ) and changes in tibiofemoral joint structures in patients with knee osteoarthritis
Source: Arthritis Res Ther. 2022 Jan 27;24:34. doi: 10.1186/s13075-022-02719-8 (PMC8793191; doi:10.1186/s13075-022-02719-8)
Supplement: Supplementary file 4 — Additional file 4: Table S4. Longitudinal associations between the morphological parameters of PTFJ and increases in tibiofemoral BMLs. [file 13075_2022_2719_MOESM4_ESM.docx]

**Supplementary Table 4.** Longitudinal associations between the morphological parameters of PTFJ and increases in tibiofemoral BMLs

|  | **Univariable** | **Multivariable*** |
| --- | --- | --- |
|  | **RR (95% CI)** | **RR (95% CI)** |
| *Increase in lateral tibial BMLs* | | |
| **Ave_COR_ang** | 1.001 (0.975,1.027) | 1.003 (0.977, 1.029) |
| **Ave_SAG_ang** | 1.014 (0.952,1.126) | 1.087 (0.958, 1.126) |
| **S** | 0.916 (0.655, 1.281) | 0.786 (0.522, 1.182) |
| **Sτ** | 0.963 (0.649, 1.429) | 0.847 (0.534, 1.343) |
| **Sφ** | 1.150 (0.789, 1.858) | 0.830 (0.380, 1.813) |
| **Sυ** | 0.828 (0.498, 1.377) | 0.659(0.358,1.211) |
| *Increase in lateral femoral BMLs* | | |
| **Ave_COR_ang** | 1.011(0.981,1.042) | 1.011(0.981, 1.043) |
| **Ave_SAG_ang** | 1.029(0.983,1.078) | 1.030 (0.983, 1.080) |
| **S** | 0.911(0.612, 1.356) | 0.877 (0.538,1.430) |
| **Sτ** | 0.822(0.507,1.334) | 0.771(0.438,1.356) |
| **Sφ** | 1.348(0.536,3.390) | 1.410(0.554,3.653) |
| **Sυ** | 0.921(0.539, 1.897) | 0.919(0.439, 2.126) |

***Adjusted for age, sex, height, weight, tibial plateau bone area, ROA, and intervention.**

**Abbreviations:**

PTFJ, proximal tibiofibular joint; BMLs, bone marrow lesions; Ave_COR_ang, the average angles of PTFJ in coronal plane; Ave_SAG_ang, the average angles of PTFJ in sagittal plane; S, contacting area of PTFJ; Sτ, load-bearing area of PTFJ; Sφ, lateral stress-bolstering area of PTFJ; Sυ, posterior stress-bolstering area of PTFJ; ROA, radiographic osteoarthritis.
